# Supplementary material for: Towards integrated metatronics: a holistic approach on precise optical and electrical properties of Indium Tin Oxide
Source: Sci Rep. 2019 Aug 2;9:11279. doi: 10.1038/s41598-019-47631-5 (PMC6677887; doi:10.1038/s41598-019-47631-5)
Supplement: Supplementary file 1 — Supplementary Info: Towards integrated metatronics: a holistic approach on precise optical and electrical properties of Indium Tin Oxide [file 41598_2019_47631_MOESM1_ESM.docx]

Towards integrated metatronics: a holistic approach on precise optical and electrical properties of Indium Tin Oxide

Yaliang Gui^1^, Mario Miscuglio^1^, Zhizhen Ma^1^, Mohammad T. Tahersima^1^, Rubab Amin^1^, Hamed Dalir^2^, and Volker J. Sorger^1,*^

^1^Department of Electrical and Computer Engineering, George Washington University, Washington, DC 20052, USA

^2^Omega Optics, Inc. 8500 Shoal Creek Blvd., Bldg. 4, Suite 200, Austin, Texas 78757, USA

[*sorger@gwu.edu](mailto:*sorger@gwu.edu)

# Ellipsometry fitting detail

In the following section we provide an accurate description of the method used for fitting the ellipsometry data using a model (Cauchy, Drude, Lorentz and Cody-Lorentz) and suitable algorithm and figure of merit, which allowed to determine fundamental parameters of the investigated film, optimizing the goodness of the fit.

1. *Cauchy model*

In our work, we use Cauchy model to fit the transparent region and find out the thickness of the thin film according to the following:

$n\left( \lambda\right)=A+\frac{B}{\lambda^{2}}$+$\frac{C}{\lambda^{4}}$

where A, B, C are adjusted to fit the refractive index for this region. Since Cauchy model is not constrained by Kramer-Kronig relation, the un-physical shape should be neglected. The advantage of Cauchy model is that it has only 2-3 free parameters are needed to achieve the fitting. But the limitation is that it can only be used in transparent region.

1. *B-spline*

We used the B-spline to expand the fitting wavelength. This kind of fitting was unrelated to the physics involved and exploited a nth degree polynomial:

$$P_{m}\left( x \right)=a_{m}x^{m}+a_{m-1}x^{(m-1)}+\ldots+a_{1}x+a_{0}$$

B-spline is designed to best match the known shape of optical constant in whole range, while Cauchy model can only describe the transparent region. Comparing with GenOsc model, B-spline can describe more optical function shapes.

1. *GenOsc model*

For addressing peaks in absorption at resonant frequencies where the material is most likely to absorb the incoming light of that wavelength, we use Genosc model. In GenOsc model, oscillator equations are used to describe resonant absorption. The permittivity function can be described as:

$$\mathfrak{I}\left( \varepsilon\left( f \right) \right)\mathfrak{=I}\left( \varepsilon_{\mathrm{Drude}} \right)\mathfrak{+I}\left( \varepsilon_{\mathrm{Lorentz}} \right)\mathfrak{+I}\left( \varepsilon_{Cody-Lorentz} \right)$$

which consists of the summation of the imaginary part of a Drude oscillator function, matching the lower frequency, the Lorentz oscillator function and two Cody Lorentz oscillator functions matching the higher frequency peak.

*– Drude oscillator.*

Drude oscillator is based on the classical equations of motion of an electron in an optical electron in an optical electric field, and fives the simplest theory of the optical constants. The Drude model portion [1] of complex permitivity is:

$$\varepsilon_{Drude, Ellipsometry}= \frac{\hbar}{\varepsilon_{0}\rho(\tau E^{2}-i\hbar E)}$$

*where* $\hbar$ *is the reduced Planck’s constant, e0 is the free space permittivity and E is light energy.*

*– Lorentz oscillator.*

Lorentz oscillator is characterized by board absorption and it is suitable for describing metal. They are used to model excess absorption near the bulk plasma frequency besides Drude model. The portion of Lorentz oscillator [2] can be described as:

$$\varepsilon_{Lorentz, Ellipsometry}= A_{1}\frac{f_{r}f_{c}}{{f_{c}^{2}-f}^{2}-if_{r}f)}$$

where $A_{1}$ is the unitless amplitude of the oscillator, fr and fc represent the broadening and central frequency of the oscillator.

*–* *Cody-Lorentz oscillator [3]*

Both Tauc-Lorentz and Cody-Lorentz can be used to describe UV region of ITO thin film.In this work, we use Cody-Lorentz. The portion of Cody Lorentz oscillator can be described as:

$$\varepsilon_{2}\left( E \right)=\frac{E_{1}}{E}e^{{(E-E_{t})}/{E_{u}}} , 0<E\leq E_{t}$$

$$\varepsilon_{2}\left( E \right)=G\left( E \right)L\left( E \right)=\left[ \frac{{(E-E_{g})}^{2}}{{(E-E_{g})}^{2}+{E_{p}}^{2}} \right]\left[ \frac{{AE}_{0}\Gamma E}{{(E^{2}-E_{0}^{2})}^{2}+\gamma^{2}E^{2}} \right], E>E_{t}$$

where $E_{1}=E_{t}G(E_{t})L(E_{t})$*.*

# RESULT DETAIL

1. **Thickness as function of the oxygen-flow rate**

Figure S1 ITO film Thickness as function of the Oxygen flow-rate (0,5,10,20,30) sccm for annealed and not annealed samples.

*Figure S2 ITO thickness as function of the oxygen flow rate (0, 5, 10, 30) sccm for different deposition time*

The deposition rate is exponentially decaying as function of the Oxygen flow rate according to the following formula: $t={ae}^{-bx}$

being $t$ the thickness, $a$=509nm the y-intercept and $b$=0.047sccm^-1^ the decay constant (RMS 97%)

1. **Spectral response (n,k) for different annealed samples batches evaporated at different time (500s, 1000s, 2000s)**

For each annealed ITO thin film, gradient structures are observed by ellipsometry. In this section, the optical constants shown below are of middle layers.

## **Deposition time=500s Argon flow-rate=40 sccm O_2_ flow-rate=0,5,10,30 sccm**

Figure S2 Spectral response of the ITO film deposited for 500s with different oxygen flow-rate conditions. No significant difference among the replicas can be found. n (left-axis) and k (right-axis) spectral analysis is carried out by spectroscopic ellipsometery. The label in the legend refers to 500s=time, 40 Argon=flow-rate and different oxygen flow-rate in the deposition chamber (0,5,10,30 sccm, in different plots) and different replicas (01-06, within the same plot)

## **Deposition time=1000s Argon flow-rate=40 sccm O_2_ flow-rate=0,5,10,30 sccm**

Figure S3 Spectral response of the ITO film deposited for 1000s with different deposition time. No significant difference among the replicas can be found. n (left-axis) and k (right-axis) spectral analysis is carried out by spectroscopic ellipsometery. The label in the legend refers to 1000s=time, 40 Argon flow-rate and analysis is carried out oxygen flow-rate in the deposition chamber (0,5,10,30 sccm in different plots) and different replicas (01-06, within the same plot)

## **Deposition time=2000s Argon flow-rate=40 sccm O2 flow-rate=0,5,10,30 sccm**

Figure S4 Spectral response of the ITO film deposited for 2000s with different deposition time. No significant difference among the replicas can be found. n (left-axis) and k (right-axis) spectral analysis is carried out by spectroscopic ellipsometery. The label in the legend refers to 1000s=time, 40 Argon flow-rate and different flow-rate in the deposition chamber (0,5,10,30 sccm in different plots) and different replicas (01-06, within the same plot)

## **ENZ Positon: Drude model fitting**

The position of ENZ is found by properly fitting using a Drude model, focusing the analysed window to the NIR region of the $\boldsymbol{\epsilon}'$ spectral response measured by ellipsometry. The x-axis intercept is considered to be ENZ position.

## ****

Figure S5 Measured real part of the permittivity $\boldsymbol{\epsilon}'$for ITO films deposited for 1500 s with different oxygen flow-rate. The labels indicate the x-intercept which is the wavelength at which ENZ corresponds. Drude-based fitting was used for extending the area beyond the measuring window.

**Spectral response (n,k) and oxygen flow-rate dependency for films deposited using 50sccm Ar flow-rate during deposition**

Film deposited for 2000s using a 50 sccm argon flow-rate display a monotonous increase of the absorption in the near infrared region as function of the an oxygen flow-rate, here modulated between 0 and 30 sccm.

## **Deposition time=1000s Argon flow-rate=50 sccm O2 flow-rate=10 sccm**

Figure S5 Spectral response of the ITO film deposited for 1000s for different oxygen flow-rate . No significant difference among the replicas can be found. The legend can be read as n (left) and k (right) and the label refers to 2000s=time, 40 Argon flowrate and different concentration of flow-rate in the deposition chamber (0,5,10,30 sccm)

**Reference**

1. Cleary, J. W., Smith, E. M., Leedy, K. D., Grzybowski, G. & Guo, J. Optical and electrical properties of ultra-thin indium tin oxide nanofilms on silicon for infrared photonics. Opt. Mater. Express 8, 1231–1245, DOI: 10.1364/OME.8.001231 (2018).

2. Zhang, C. et al. Robust Extraction of Hyperbolic Metamaterial Permittivity Using Total Internal Reflection Ellipsometry. ACS Photonics 5, 2234–2242, DOI: 10.1021/acsphotonics.8b00086 (2018).

3. Fujiwara, C. R. W., Hiroyuki. Spectroscopic Ellipsometry for Photovoltaics (Springer International Publishing, 2018).
